# Inferring disease transmission networks at a metapopulation level

**DOI:** 10.1186/2047-2501-2-8

**Published:** 2014-11-17

**Authors:** Xiaofei Yang, Jiming Liu, Xiao-Nong Zhou, William KW Cheung

**Affiliations:** Department of Computer Science, Hong Kong Baptist University, Hong Kong SAR, China; National Institute of Parasitic Diseases, China CDC, Shanghai, China

**Keywords:** Network inference, Disease transmission networks, Metapopulation, Bayesian learning

## Abstract

**Background:**

To investigate transmission patterns of an infectious disease, e.g., malaria, it is desirable to use the observed surveillance data to discover the underlying (often hidden) disease transmission networks. Previous studies have provided methods for inferring information diffusion networks in which each node corresponds to an individual person. However, in the case of disease transmission, to effectively propose and implement intervention strategies, it is more realistic and reasonable for policy makers to study the diffusion patterns at a metapopulation level when the disease transmission is affected by mobile population, that is, to consider disease transmission networks in which nodes represent subpopulations, and links indicate their interrelationships.

**Results:**

A network inference method called NetEpi (Network Epidemic) is developed and evaluated using both synthetic and real-world datasets. The experimental results show that NetEpi can not only recover most of the ground-truth disease transmission networks using only surveillance data, but also find a malaria transmission network based on a real-world dataset. The inferred malaria network can characterize the real-world observations to a certain extent. In addition, it also discloses some hidden phenomenon.

**Conclusions:**

This research addresses the problem of inferring disease transmission networks at a metapopulation level. Such networks can be useful in several ways: (i) to investigate hidden impact factors that influence epidemic dynamics, (ii) to reveal possible sources of epidemic outbreaks, and (iii) to practically develop and/or improve strategies for controlling the spread of infectious diseases.

## Background

Infectious diseases such as influenza and H1N1 are transmitted between individuals. This process has been widely studied by researchers in biology, statistics, epidemiology, public health, etc. for many years. Their objectives are to help front-line practitioners and policy makers to control disease outbreaks and to prevent severe morbidity and mortality. Various intervention strategies have been applied, including but not limited to vaccination, contact deduction, etc.

Another strategy, contact tracing, is also widely used to prevent disease outbreaks [1]. It is a network-based approach conducted at an individual level. Susceptible individuals are identified and monitored to minimize the chances of infection. The network-based approach not only differentiates individuals in host populations [2] but also allows for performing individual-level simulations [3]. This approach is similar to the one adopted in the research on tracing the transmission pathways of infectious diseases, e.g., malaria, particularly the disease transmission is affected by mobile population, except that here disease transmission is examined at a metapopulation level. Nodes and edges within the metapopulation-based disease transmission networks do not represent individual persons and their pairwise connections (e.g., social contacts [4]); instead, they represent patches of subpopulations (e.g., provinces, cities, and townships) and various transmission pathways among them (e.g., highways and air travel routes). Both individual-based and metapopulation-based studies of disease transmission networks are useful in the following aspects:

- Analyzing epidemic phase transition behavior [5];
- Investigating the dominant factors that underlie the spread of a disease epidemic [6];
- Providing suggestions to effectively control epidemics by cutting off transmission pathways and/or isolating certain local regions [7].

Many of the existing disease transmission studies that deal with the above two types of transmission networks share the similar limitation, that is, they assume that network structures are given in advance; for example, contact structures for influenza spreading [8, 9] or airlines for the spread of H1N1 [10] and SARS [11]. In these studies, information about which person or location will be infected is given. However, in an actual epidemic, only the spatiotemporal surveillance datasets containing the infection times and locations of reported infection cases are obtained [12]. This type of data provides no knowledge of the hidden transmission pathways that denote the routes of disease propagation among geographical locations. This real-world situation poses a significant and undeniable challenge to policy makers who are responsible for applying intervention strategies at appropriate times and locations. In this regard, inferring disease transmission networks becomes an important and urgent research problem in epidemiological studies (as in [13]).

The network inference problem has been recently and widely studied in the research domain of information diffusion. Based on empirical time-series data that indicates when people become informed or infected, the static network inference problem with a homogeneous edge setting (edge weights are the same for the whole ground-truth network) can be transformed into a combinatorial optimization problem [14]. By formatting it as an MAX-*k*-COVER problem, Gomez-Rodriguez et al. have proven that selecting the top *k* edges that maximizes the likelihood of the static network structure is NP-hard. Therefore, they introduced a greedy algorithm based on the submodularity property [15] to approximate an optimal solution. A similar problem with heterogeneous edge weights was formulated into a convex optimization problem, and a maximum likelihood method was proposed to solve it [16]. In doing so, noticing that the structure of a social network is sparse, Myers and Leskovec introduced penalty functions into the objective function to improve its accuracy [16]. The same problem was further extended from inferring static network structures to inferring dynamically changing networks, and the effect of a time-varying external influence was integrated into the model [17]. Recently, to infer disease transmission networks at an individual level, Teunis and Heijne defined a pairwise kernel likelihood function to incorporate infection time difference, proximity of cases, and genetic similarity information [18].

Although information diffusion and disease transmission are to a certain extent similar, they have significant practical differences. Information diffusion networks are usually analyzed at an individual level, whereas disease transmission networks are more meaningful and practical if analyzed at a metapopulation level, for the following reasons:

- It is more appropriate to simulate disease transmission in both temporal and spatial scales [19, 20].
- It is difficult to simulate complex individual human behavior and collect large amounts of personal information [6, 21, 22].
- Controlling disease transmission at a metapopulation level is more practical from the view point of front-line practitioners and policy makers [23].

However, the metapopulation approach leads to two additional challenges:

1. Nodes within metapopulation-based disease transmission networks connect not only with each other, but also to themselves, indicating that susceptible people may get infected by infectious people within the same subpopulation.
2. Unlike information diffusion or individual-based disease transmission networks, disease transmission at a metapopulation level does not follow Directed Acyclic Graphs, where if certain individual does not get informed or infected at the first time, he or she will never get informed or infected in the following time period. In contrast, it propagates over Directed Cyclic Graphs. That is to say, a subpopulation may repeatedly get infected as long as it contains susceptible people. In such transmission network, disease proceeds with cyclic loops rather than like a path or branches of trees.

In such a situation, inferring metapopulation-based disease transmission networks is not only desirable but also challenging. Currently, to the best of our knowledge, no such studies exist. Specifically, this research makes the following three contributions:

1. A generalized linear disease transmission model is built, which considers all the possible transmission pathways at a metapopulation level.
2. A machine learning method called NetEpi (Network Epidemic) is developed to infer hidden disease transmission networks using only the spatiotemporal surveillance data.
3. Unlike similar network inference studies which are conducted over Directed Acyclic Graphs, the proposed method addresses the problem over Directed Cyclic Graphs when analyzing real-world situations.

This research is also practically meaningful as it helps to computationally predict the spread of infectious diseases and provides policy makers with new insights with potentially effective intervention strategies [20]. Partial results of this research have been reported in [24, 25].

## Method

### Definitions

Suppose there exists an unknown directed cyclic network *G* over which an infectious disease transmits, the observed surveillance data can be represented in a tuple of <*id*_*p*_,*it*_*p*_,*loc*_*p*_>. *p* is the index of a reported/confirmed case. *id*_*p*_ represents the unique identity. *it*_*p*_ is the reported infection time. *loc*_*p*_ is the geographical location where the reported/confirmed case *p* gets infected.

After aggregating infection cases based on locations and infection times, a dataset *D* = {<*v*_*i*_,*ic*_*i*_,*t*_*i*_> | *i* = 0,1,2,…*N*,*t*∈*T*} is collected. *i* is the index of a specific node. *v*_*i*_ corresponds to the unique identity of a geographical location (e.g., a province, a city, a township, or an urban area). *ic*_*i*_ is the aggregated number of infection cases. *t*_*i*_ indicates a time step. *T* is the considered time period of disease transmission. In this research, given only the observed data *D*, the underlying disease transmission network *G* is inversely inferred. The estimated disease transmission network is referred to as *G*^∗^.

*Definition 1. Disease Transmission Network*: Graph *G*=<*V*,*E*> is a directed cyclic network where *V* = {*v*_*i*_ | *i* = 0,1,2,…,*N*} is the set of nodes. The node *v*_0_ represents the source node of the imported cases that would potentially cause local epidemics (the imported cases for a disease can be defined as the laboratory-confirmed infection cases where people have traveled to disease endemic regions or countries within days before the onset of the disease [26]). *v*_*i*_ (*i* = 1,2,…*N*) correspond to the rest of nodes within the target region. *E* = {***e***_*i*_ | *i* = 1,2,…,*N*} denotes the set of directed edges with different weights *W* = {***w***_*i*_ | *i* = 1,2,…,*N*}. ***e***_*i*_ = {*e*_*ji*_ | *j* = 0,1,2,…,*N*} is the set of incoming links for node *i* and ***w***_*i*_ = {*w*_*ji*_ | *j* = 0,1,2,…,*N*} is the corresponding weight vector. To be noticed, the source node *v*_0_ does not have incoming links. The physical meanings of these edges that have non-zero weights can be understood as the generalized transmission pathways that *temporally correlate* subpopulations in terms of their infection observations.

**Figure 1:**
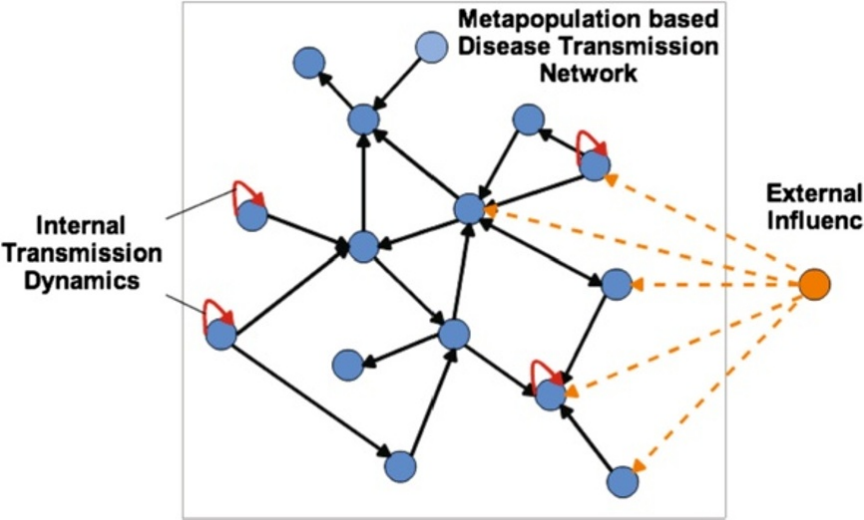
**An illustration of three types of transmission pathways contained in our considered disease transmission networks.** The internal transmission component is labeled with red solid links connecting to the nodes themselves. The neighborhood transmission component is labeled with black solid links between nodes within the metapopulation-based disease transmission network. The external influence component is introduced as dashed orange links (an external node connects to all the other nodes; for the sake of presentation, we draw only a proportion of them).

**Figure 2:**
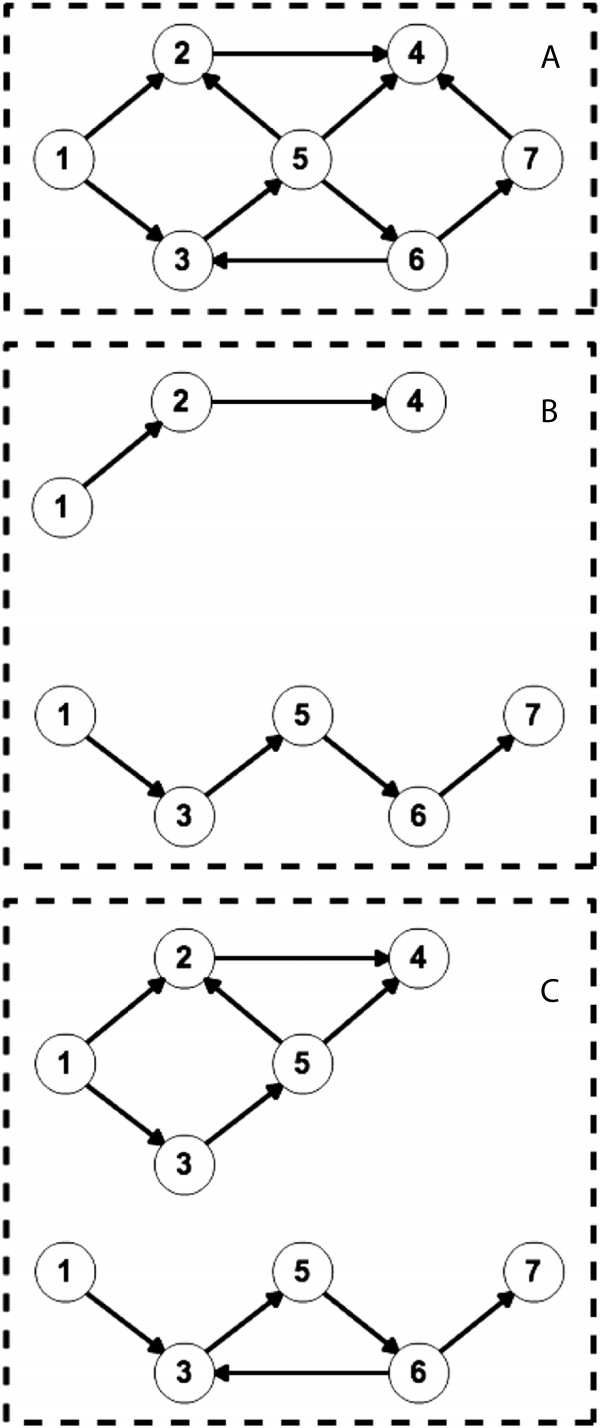
**Differences between information diffusion and disease transmission over the same directed cyclic network.** **(A)** shows the example of a ground-truth synthetic network. **(B)** shows two independent information cascading or individual-based disease transmission processes where no cycle exists in these processes. **(C)** shows two independent disease transmission processes at a metapopulation level.

Unlike the network structures used in previous studies, the network structures used in this research contain three types of transmission pathways (shown in Figure 1). As the data describes a real-world situation, the assumption is that infected people can infect susceptible people within the same subpopulation (shown in Figure 2). This type of transmission pathway is defined as the internal transmission component. In addition, subpopulations within metapopulation-based disease transmission networks can be affected not only by subpopulations located in adjacent geographical regions, but also by imported cases. We define them respectively as the neighborhood transmission component and the external influence component.

*Definition 2. Internal Transmission Component*: Within each node (subpopulation), previously infected people may correlate to newly infected people without outside disturbances. This component is disease independent. Air-borne diseases such as influenza, vector-borne diseases such as malaria, and other infectious diseases all have this property. It is represented as an edge linking to itself with weight *w*_*ii*_ for each node *i* in the disease transmission network *G*.

*Definition 3. Neighborhood Transmission Component*: Among groups of nodes (subpopulations), the temporal correlations among infected people could be caused by physically connected highways, air travels, adjacent borders, etc. This component signifies the interactions happening between infected people in different subpopulations. In *G*, it is represented as a directed link *e*_*ij*_ from nodes *i* to *j* with weight *w*_*ij*_, indicating the correlations between infected people in both *i* and *j*.

*Definition 4. External Influence Component*: In disease transmission, the imported cases from foreign or distant endemic countries and regions are another major factor that can cause local epidemics [27]. Thus, we consider this factor in the disease transmission network as an external node connected to all the other nodes. In *G*, this is denoted as an edge *e*_0*i*_ from external node to node *i* with weight *w*_0*i*_.

### Linear transmission model

To characterize a disease transmission process over *G*, we integrate both of the internal transmission component and the external influence component with the neighborhood transmission component. The internal transmission component characterizes the possible transmission relationships between previously infected people and current infected people within each subpopulation. The assumption in [19], that “individuals do not change disease states during movements” is retained. Thus the neighborhood transmission component describes the temporal correlations between infected people in different subpopulations. The external influence component depicts the introduction of the imported cases from external sources. The above three types of transmission pathways are defined in mathematical forms, respectively, as follows:
1

where *itc*_*i*_^*t*^, *ntc*_*i*_^*t*^, and *eic*_*i*_^*t*^ refer to the number of infection cases from the internal transmission, neighborhood transmission, and external influence components of node *i* (*i*≠0) at time step *t*, respectively. *N*_*i*_ is the number of the neighbors of node *i*. *w*_*ii*_, *w*_*ji*_, and *w*_0*i*_ are the corresponding edge weights. *ic*_*i*_ is the total number of infection cases in node *i*, which can be written as a linear combination of the above three components plus an error term *ε*. *ε* is used to capture unpredicted biases. The assumption is that the infection number for each node follows a zero-mean normal distribution, *ε*∼*N*(0,*β*):
2

Equations 1 and 2 characterize the temporal dynamics of the infection cases at each location. Note that in the real world, once a reported/confirmed case is diagnosed, the physicians or hospitals would take necessary treatment and intervention measures, for example, medication or isolation. Thus, in the above linear transmission model, the infection cases at the current time step would be set to be isolated in the subsequent time steps.

### Network inference problem

The network inference problem to be solved here is how to inversely infer the existence of the edges within the hidden disease transmission network *G* and their corresponding weights *W*={***w***_*i*_ | *i*= 0,1,2,…,*N*}, given an observed surveillance dataset *D*={<*v*_*i*_,*ic*_*i*_,*t*_*i*_> | *i*=0,1,2,…,*N*,*t*∈*T*}. Since the disease transmission process at the metapopulation level does not follow the Directed Acyclic Graphs pattern (Figure 2), it would be inaccurate to infer disease transmission networks following the cascading process in the information diffusion [14].

To recover the network structure *G*, it is necessary to first write the likelihood function for a specific node *i* based on Eq. 2:
3

where all the parameters are the same as those in Eq. 2, except we use , and  rather than *N*_*i*_, to indicate the number of estimated neighbors of node *i* within the inferred network *G*^∗^. *β* is the variance of the normal distribution for the error term *ε*. Based on this equation, we transform the network inference problem into an optimization problem, which is to find the optimal combination of neighbors with accurate weights for a specific node *i*.

Then for the entire estimated network *G*^∗^, the objective is to maximize the likelihood function:
4

To evaluate the estimated network *G*^∗^, we will use precision-recall measures. Specifically, we will compare both the existences of edges and their corresponding weights in the synthetic network *G* and the estimated network *G*^∗^.

### Partial correlation network construction

Because there could be many combinations for a node to form its neighborhood, the solution space for the above problem is huge. At the first step, we plan to reduce this space in order to improve both accuracy and performance for further tuning.

**Figure 3:**
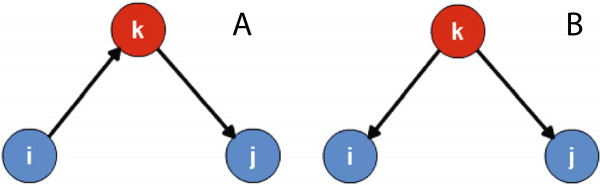
**The possible transmission relationships among three nodes** [28]**.** The blue ones are the target nodes for which we aim to identify their relationships. The red nodes are the intermediate nodes. **(A)** shows no directed edge between nodes *i* and *j*. The disease transmission follows a path from node *i* to the intermediate node *k*, then to the target node *j*. **(B)** shows that node *k* transmits to nodes *i* and *j*, simultaneously and independently.

When using the Pearson correlation to analyze the correlation between two selected nodes *i* and *j*, a problem arises in the analysis of disease transmission networks. As shown in Figure 3(A), disease transmission may follow a path from node *i* to *k*, then to *j*. Take nodes *i* and *j* as our analysis targets. Although they are not directly connected, and the overall time-series surveillance data exhibits time delay, they may still be correlated. Therefore, in the approximate network structure *G*^*p*^, they may be connected. The same problem exists in the case of Figure 3(B), where both nodes *i* and *j* are the children of node *k* in the disease transmission process. The correlation between nodes *i* and *j* is still strong even though the weights *w*_*ki*_ and *w*_*kj*_ are very different. To solve the biases produced by the intermediate node and the sharing of the same parent node, a first-order partial correlation analysis is carried out.

The first-order partial correlation is a measurement of the dependence between two variables X and Y, after removing or fixing a third variable Z. In our case, to compute it between nodes *i* and *j*, the effect of another node *k*, where *k*=0,1,2,…,*N*, and *k*≠*i*,*j* is sequentially removed or fixed. From the results, only those coefficients that indicate strong correlations with significant p-values are chosen. It should be mentioned that a partial correlation analysis usually does not provide edge direction information [28, 29]. Therefore, to infer a directed relationship, in this research, we analyze the partial correlation with a time lag. The physical meaning of the time lag is a time step during the disease transmission process (e.g., one day, one week, or one month). Here, we use a time lag of one unit as example, but the time lag is not limited to one unit, other options are also allowed. The direction is defined as from the node using the previous-time-step time-series data to the node using the current-time-step time-series data. Defining the partial correlation coefficient between nodes *i* and *j* after fixing the variable of node *k* as *ρ*_*ij*.*k*_, it can be computed as follows:
5

where *ρ*_*ij*_, *ρ*_*ik*_ and *ρ*_*jk*_ are the covariances between each pair of node *i*, *j* and *k* respectively.

### Back-tracking Bayesian learning

Given the partial correlation network *G*^*p*^, an approximate disease transmission network structure is obtained that contains possible neighbors for each node. However, some edges in *G*^*p*^ still do not exist in the synthetic network *G*. A possible solution is to set the weights of these false positive edges within *G*^*p*^ as zero during the inference process. This is similar to the procedure of removing irrelevant basis components, which is the basis for dimension reduction [30]. In the proposed inference method, the Bayesian learning is based on the Sparse Bayesian Learning (SBL) framework [31]. Related work has been widely and well reported in signal processing studies [30]. To be noticed, if two components are similar, SBL only chooses one of them in order to compress the relevant information. However, in our case, even two nodes are similar, we aim to find both of them.

For a specific node *i*, the preprocessed surveillance dataset *D* is divided into two subsets: an *M*×1 vector of ***y***={<*v*_*i*_,*ic*_*i*_,*t*_*i*_> | *t*_*i*_ = 2,3,…,*M*+1,*M*∈*T*} and a *M*×|*N*^*p*^| matrix of ***x*** = {<*v*_*j*_,*ic*_*j*_,*t*_*j*_> | *j*∈*N*^*p*^,*t*_*j*_ = 1,2,…,*M*,*M*∈*T*-1}. *M* is the size of output variable ***y*** and input variable ***x***. *N*^*p*^ represents the indices of the possible neighbors that node *i* has based on *G*^*p*^. *T*-1 is the previously considered time period of disease transmission. For the sake of presentation, in the following, we omit the index *i* for ***y***, ***x***, and other parameters. If not specifically stated, all the parameters are formulated for node *i*. Here, we use a time lag of 1 between ***y*** and ***x***.The relationship between ***y*** and ***x*** can be formulated based on the generalized linear transmission model introduced earlier as follows:
6

where ***w*** = {*w*_*j*_ | *j*∈*N*^*p*^} is a vector indicating the weights of all possible incoming links estimated based on *G*^*p*^. *ε* is an error term. As mentioned earlier, the solution space is huge. Thus we hope to limit ***w*** within a smooth range. Here we follow the framework of SBL, and let both ***w*** and *ε* follow a zero-mean Gaussian distribution with variances of ***α*** and *β*, respectively [31]. They are defined as:
78

Because there is no prior knowledge of ***w*** and *ε*, it is reasonable to set them with non-informative prior distributions, such as a Gamma distribution. Here, ***α*** and *β* are assumed to have the same hyperparameters for all nodes.

Given the observation data ***y*** and the prior distribution ***α*** and *β*, the posterior distribution of ***w*** is:
9

which is a Gaussian distribution *N*(***μ***,***Σ***) with
1011

where . “Type-II maximization likelihood” maximization combined with a maximum a posteriori probability (MAP) estimate [31] transforms the whole problem into the following marginal likelihood function:
12

Writing Eq. 12 into a logarithm form , we have:
13

with
14

The derivatives of Eq. 13 with respect to *α*_*j*_ and *β* are [32]:
1516

Setting Eqs. 15 and 16 to zero, the estimations of *α*_*j*_ and *β* become:
1718

The above iterative estimation procedure can be solved by using the Expectation-Maximization. In each iteration, the contributions to the marginal likelihood function are estimated for all the nodes in *G*^*p*^. The one with the maximum contribution is selected as the candidate neighbor. Its corresponding weight is then computed.

**Figure 4:**
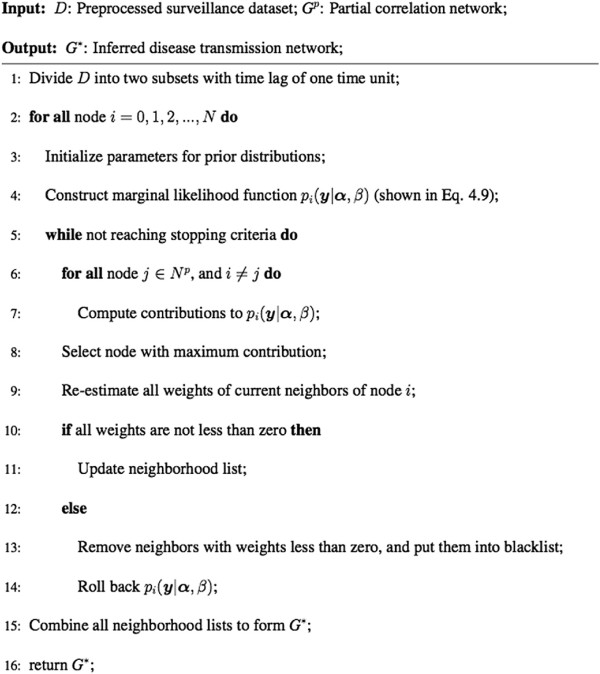
**Algorithm for the Back-Tracking Bayesian Learning.**

In the disease transmission network *G*, only positive links indicating the existence of transmission pathways exist. However, the prior distribution shown in Eq. 7 may cause ***w*** to be negative. To avoid this, a constraint limiting ***w*** to a positive value is introduced. To incorporate this constraint into the framework of the above Bayesian learning, a back-tracking technique is used. During the EM learning procedure, the marginal likelihood function and other parameters are updated sequentially. Consequently, each time ***μ***, ***Σ***, *α*_*j*_, and *β* are updated, any *α*_*j*_ that fail the constraint are selected out, and their corresponding indices are put into a blacklist. The learning procedure is then rolled back, including the marginal likelihood value, to the previous step, and proceeds by selecting only nodes that do not appear in the blacklist, while at the same time maximizing the marginal likelihood function. The algorithm for the Back-Tracking Bayesian Learning is shown in Figure 4.

## Results

### Experiments based on synthetic data

Three types of Kronecker Graphs [33] are constructed: (i) core-periphery networks, which have a cluster of nodes in the core of the network and other nodes with less connections distributed in the periphery area, (ii) hierarchical community networks in which nodes form several small communities that are connected to form one large cluster, and (iii) random graphs, which have no obvious pattern. For each type of network structure, different scale parameters are set to generate different ground-truth networks: (i) 64 nodes with 100 edges and 150 edges, (ii) 128 nodes with 180 edges and 200 edges, (iii) 256 nodes with 350 edges and 400 edges, and (iv) 512 nodes with 720 edges and 800 edges. The external links and self-connected edges are generated independently for each ground-truth network. For each synthetic network, disease transmission model (Eq. 2) is run ten times to generate independent synthetic datasets. For a single dataset, the transmission process is made to cover all the edges of *G*. In total, there are three types of network topologies × 8 different sizes × 10 independent transmission processes = 240 datasets.

**Figure 5:**
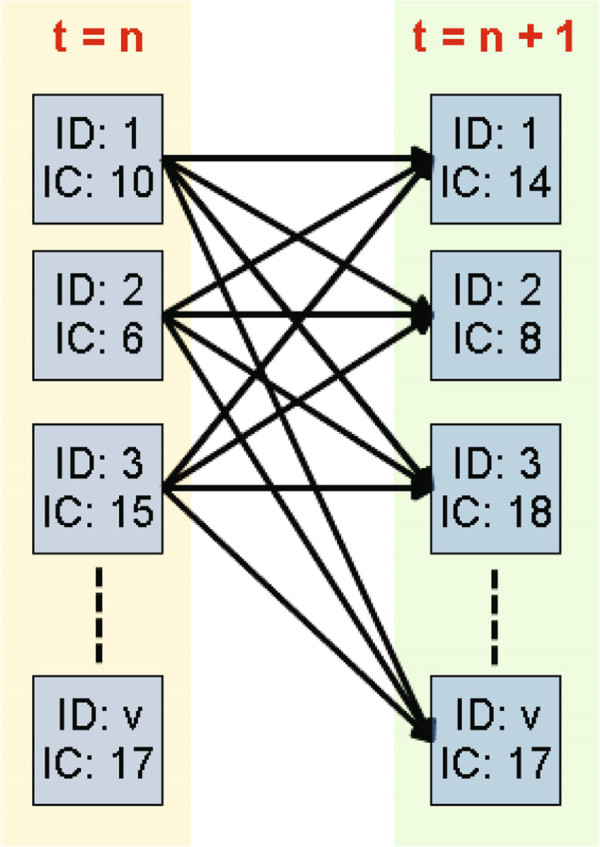
**An overview of the baseline method.** Nodes (represented as squares) have infection cases at time step *t* = *n*, and have probabilities of infecting nodes that have infection cases at time step *t* = *n*+1. This is shown as the dashed lines in the figure. The ID notation represents the unique identity number of each node. The IC notation represents the number of infection cases at the current time step.

To our best knowledge, there has not been much prior work on inferring network structures over Directed Cyclic Graphs. Therefore, we utilize a probability based baseline method. At two adjacent time steps *t* = *n* and *t* = *n*+1, all the nodes that have infection cases at *t* = *n* will have directed connections to those nodes that have infection cases at *n*+1 (shown in Figure 5). The edge weight is affected by both the number of infection cases and the number of infected nodes in the previous time step. The top *k* edges with the highest weights are selected, and the estimated disease transmission network *G*^∗^ is constructed accordingly. The mathematical formula to compute the baseline edge weight is as follows:
19

**Figure 6:**
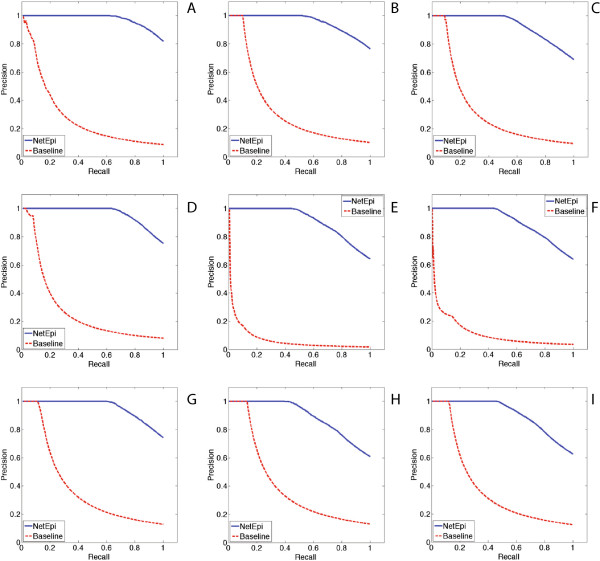
**The precision-recall curves for synthetic networks.** We test NetEpi over 24 different networks, and we select nine of them here for illustrations. **(A) - (C)** show core-periphery networks with size of 64 nodes with 100 edges, 128 nodes with 180 edges, and 256 nodes with 350 edges. **(D) - (F)** show hierarchical community networks with size of 64 nodes with 100 edges, 128 nodes with 180 edges, and 256 nodes with 350 edges. **(G) - (I)** show random graphs with size of 64 nodes with 100 edges, 128 nodes with 180 edges, and 256 nodes with 350 edges. We compare the performances of NetEpi with the baseline method. To be noticed, NetEpi outperforms the baseline method in all datasets.

To evaluate the inference results, the precision-recall curves are computed as shown in Figure 6. Similar to the definitions in [14], the precision is defined as “what fraction of edges in *G*^∗^ is also present in *G*”, and the recall is defined as “what fraction of edges of *G* appears in *G*^∗^”. For two nodes *i* and *j*, if both the ground-truth edge *e*_*ij*_ and the inferred edge  exist, and the difference in their corresponding weights  is less than a predefined threshold, we say the inferred edge is accurate. In our experiments, NetEpi outperforms the baseline method in all 240 datasets.

For a specific node in the disease transmission network, NetEpi treats all the other nodes homogeneously and independently. That is to say, the connections between two nodes *i* and *j* are only affected and estimated by the time-series surveillance data of these two nodes. This exactly satisfies the real-world requirements discussed above. The underlying network topology is not taken into account during the inference procedure. For networks that have same sizes but different topologies, NetEpi performs best on the core-periphery networks.

In core-periphery networks, nodes are located in the core region. These nodes have more connections than those distributed in the periphery region. Therefore, to achieve an optimal solution, core-located nodes will have higher probabilities of possessing many neighborhood combinations. In other words, the probability of finding a globally optimal solution for such nodes will decrease as the number of their incoming edges increases. The accuracy of NetEpi over networks with core-periphery topology is consequently biased by the tradeoff between core-located nodes and periphery-located nodes. In comparison, networks with a hierarchical communities topology do not have single cores. The single core is divided into several sub-cores that individually form sub-communities. This structure increases the average number of combinations for each node and directly affects the inference accuracy. As for the random graphs, no matter where the nodes are located, their number of connections does not have a fixed pattern. Consequently, NetEpi achieves oscillating results, which means the precision-recall results for random graphs are sometimes the best, and sometimes the worst.

Here, the out-degree is used to illustrate the accuracy differences between networks with different topologies. It is defined as follows:
20

where *d*_*i*_ is the out-degree for node *i* and *d*_*avg*_ is the average out-degree for the whole network. The out-degree statistics for all the 24 synthetic networks are listed in Table 1.

**Table 1:** **Out-degrees for synthetic networks**

Size	Core-periphery	Hierarchical	Random
	network	community	graph
		network	
64 nodes, 100 edges	1.4154	1.5385	1.6923
64 nodes, 150 edges	1.7385	1.8308	1.8923
128 nodes, 180 edges	1.3876	1.4496	1.4651
128 nodes, 200 edges	1.5504	1.6357	1.5969
256 nodes, 350 edges	1.3619	1.5175	1.5097
256 nodes, 400 edges	1.5525	1.6537	1.6615
512 nodes, 720 edges	1.4016	1.5107	1.5029
512 nodes, 800 edges	1.5439	1.6199	1.6686

**Figure 7:**
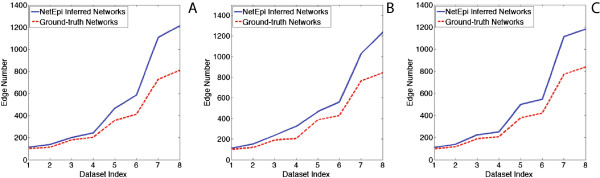
**Differences between the edge number in the inferred networks and the ground-truth networks.** For each dataset index, we take the average of all the networks with different topologies but same size. The network size increases as the index increases. **(A) - (C)** show the results of core-periphery networks, hierarchical community networks, and random graphs respectively. It is obvious that as the ground-truth network size increases, the accuracy of NetEpi decreases. The number of false edges increases as well. This results from the increased number of possible combinations of neighbors for each node to achieve its global optimal solution.

For networks with the same topologies but a different number of nodes, NetEpi achieves better results when inferring smaller networks, as shown in Figure 6. At the beginning of the inference process, no edge information is given. Therefore, a ground-truth network is treated as a complete network. Even given its approximate structure *G*^*p*^, the complexity quadratically increases as the number of nodes increases. Meanwhile, as the edge number increases, the number of neighborhood combinations needed for each node to achieve an optimal solution also increases, which directly interferes the inference results, as shown in Figure 7.

However, inferring disease transmission networks at a metapopulation level is different from inferring individual-based information diffusion networks from the perspective of network size. Network size is usually small when calculated at the administrative level (e.g., province and township levels). For example, for a global epidemic disease, WHO publishes statistical reports at the country level (e.g., dengue and malaria [34] (two types of neglected tropical disease that transmit between human and mosquitoes)); for an infectious disease such as SARS, China reports at a province level, on a daily basis. One possible method for inferring large-size networks that cross several levels is to perform hierarchical clustering. NetEpi begins the analysis at the highest level, where each node represents a cluster of lower-level nodes. Then, within each higher-level node, NetEpi can be performed again to infer the lower-level transmission networks. This whole process can be repeatedly and sequentially conduced to get a whole picture of large-size networks.

**Figure 8:**
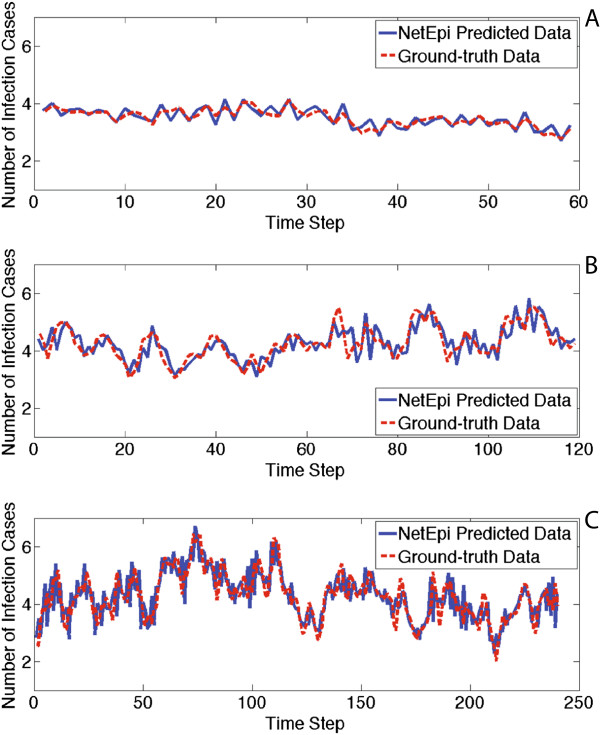
**NetEpi accurately captures the disease transmission trend.** Three synthetic networks with different topologies and sizes are selected for illustration: **(A)** shows a core-periphery network with 64 nodes and 100 edges. **(B)** shows a hierarchical community network with 128 nodes and 180 edges. **(C)** shows a random graph with 256 nodes and 350 edges.

Experiments show that all the predicted epidemic trends that occur in the ground-truth networks are captured by the inferred networks, no matter how large the networks are. Because of the space limitation, here we show some examples in Figure 8. This confirms that NetEpi converges to a optimal solution, although it may not be the global one.

### Experiments based on real-world data

The real-world dataset was provided by the Chinese Center for Disease Control and Prevention. It contains the reported malaria infection cases in Yunnan province, China. Two types of cases, infected by two distinct types of malaria parasites, (*Plasmodium falciparum, and Plasmodium vivax*), are mixed together. Here, the focus is on *Plasmodium vivax*, which is the dominant type in the Yunnan region. There were 2928 cases reported in 51 townships in 2005. These townships are distributed along the border between China and Myanmar (a high malaria-endemic country). The data are preprocessed by merging those cases reported in the same townships and filtering out those infected with another type of malaria parasite that is not the focus in this research. These townships are further classified into five categories based on their disease severities. They correspond to different numbers of infection cases during the year and are labeled with different colors: (200,+*∞*) (red node), (150,200] (purple nodes), (100,150] (green nodes), (50,100] (yellow nodes), and (0,50] (blue nodes).

The dataset is sparse, with missing data. Moreover, there is no complete labels indicating the imported cases or information about the sources that introduce the imported cases in the original surveillance dataset. Thus, a fixed external node could not be set up during the inference procedure. Like the periodical pattern of the Internal Transmission Component, the External Influence Component also presents regular pattern because of the frequent human mobility motivated by cross-border trade and business. We consequently merge EIC with ITC, and represent either of them, or their combination, by self-connected edges. This is reasonable because it has been recorded that most of these imported cases were due to working, trading, and/or visiting in/with Myanmar regularly. Therefore, self-connected edges are able to capture these regular patterns and identify the imported cases. It is expected that there are many cases imported from neighboring countries, especially Myanmar; therefore, the inferred malaria transmission network contain many self-connected edges. It has been widely reported that the incubation time for *Plasmodium vivax* is 12∼17 days [35]. However, studies have also reported that the incubation time can be longer, from several months to several years [35, 36]. Therefore, in this research, 21 days has been selected as the time window for inferring the hidden malaria transmission network.

In the inferred malaria transmission network, the self-connected edges are labeled with dashed red lines, and edges between neighboring nodes are linked with solid black lines. The width of the edges indicates the strength of the transmission pathways. There are basically two classes of nodes. Some of them connect to themselves, as expected, whereas others form two small communities. In the following, the two types are interpreted separately.

**Figure 9:**
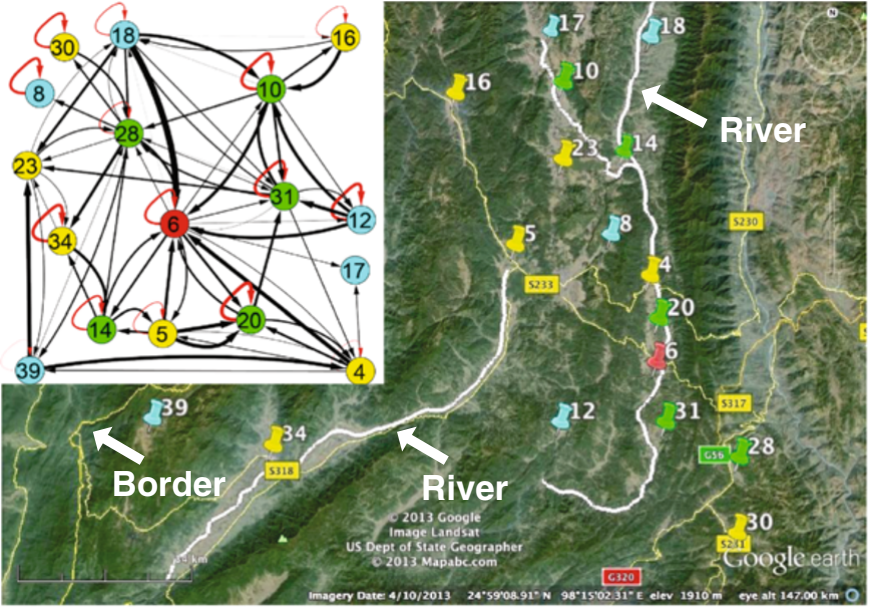
**Townships that form a big community as shown in the upper-left subfigure are correlated by their locations that are distributed either in the upstream and downstream of rivers, or close to the highways that connect each other.**

**Figure 10:**
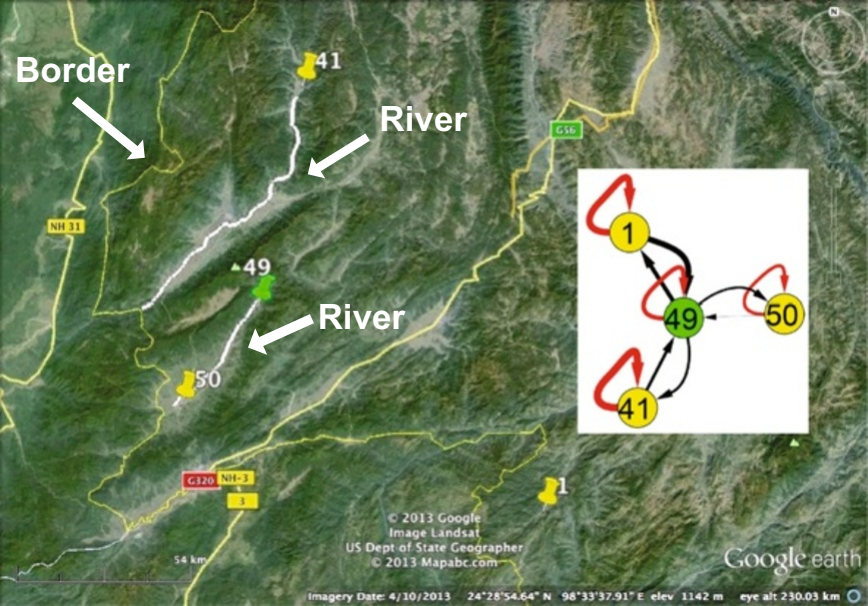
**Townships in this figure are located relatively far from each other, except 49 and 50.** Their connections may result from sharing the same source of the imported cases.

*Small Communities:* Figures 9 and 10 show that there are two communities in the whole malaria transmission network. The larger one (Figure 9) includes the nodes with the most severe epidemic situations. The severest township, 6, has connections to all the other second-level severity townships (green nodes), indicating that their disease transmission interactions may be the dominant reason for the local malaria endemics in the region. It is obvious that most nodes are connected by highways (e.g., S231, S233, S317 and S318) and rivers. The highways allow infectious patients to move among subpopulations, thus increasing the exposure risk of susceptible populations. The river usually plays a significant role in malaria endemics. It provides a suitable environment for the vector of malaria to reproduce and its flow moves the larva of vector downstream. Therefore, it is possible that the endemics within townships are affected by internal malaria transmission dynamics.

**Figure 11:**
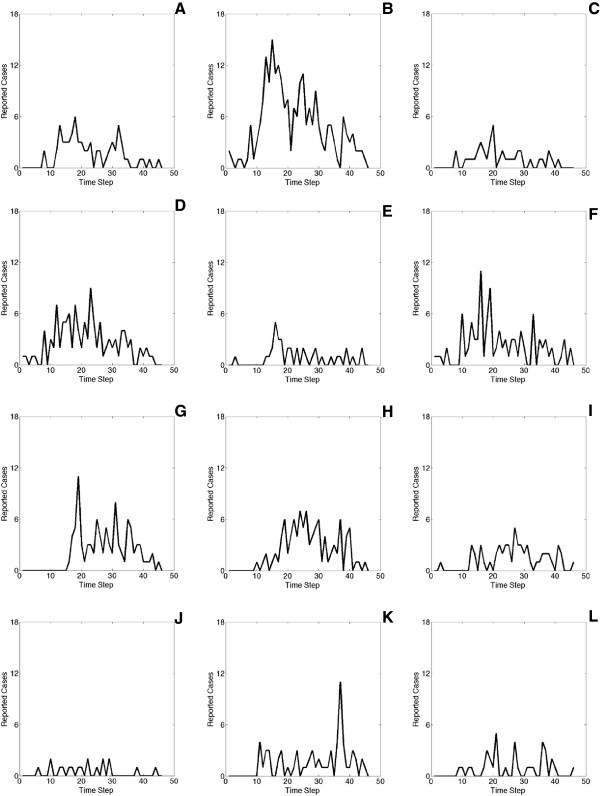
**The reported cases for the selected nodes in 2005.** In order to present them clearly, we aggregate the reported cases on an eight-day basis. **(A) - (F)** show the curves for townships selected from Figure 9. Their administrative names are Shangying Township, Wuhe Township, Beihai Township, Qushi Township, Jietou Township, and Zhenan Township, respectively. **(G) - (I)** show the curves for townships selected from Figure 10. Their administrative names are Mengdui Township, Qingping Township, and Zhangfeng Township, respectively. **(J) - (L)** show the curves for townships selected from Figure 12. Their administrative names are Tongbiguan Township, Mengyue Township, and Longba Township, respectively.

It can readily be noted from Figure 9 that some inferred edges are thicker than others, denoting higher transmission influences (larger edge weights). *e*_18-6_ (the dash in the index is used for separation) is much thicker than the others, for example, *e*_14-6_, *e*_4-6_, and *e*_28-6_. We interpret this based on Figure 11(A)-(F) in which reported cases are aggregated on an eight-day basis for clear presentation. As shown, although township 18 (Figure 11(E)) has fewer reported cases than other example townships and contains many zero-case intervals, its temporal trend does not significantly violate the trend of township 6 (Figure 11(B)). In comparison, the “mountain-valley-mountain” pattern of township 6 can only be partially matched with other townships (e.g., townships 4 (Figure 11(A)), 14 (Figure 11(D)) and 28 (Figure 11(F))). The influence from township 6 to 4 is much less than that from the reverse direction. This is because the second highest peak appearing between time step 20 to 30 in the trend of township 6 cannot contribute to the valley appeared at the same time interval in the trend of township 4. However, the reverse contribution is reasonable. Intuitively, the pair of townships 4 and 8 (Figure 11(C)), and the pair of townships 14 and 28 have similar trends respectively, but NetEpi only finds edges between townships 14 and 28. This is due to that, for townships 4 and 8, their trends before time step 20 seem to be similar, but those after step 20 present a time lag of around 8*8 days.

As for the small community, it contains townships 1, 41, 49 and 50. The distance between townships 1 and 49 is much longer compared with that between townships 50 and 49. In addition, townships 49 and 50 share the same river. However, the relationships between 49 and 50 are much weaker than those between 1 and 49. It is speculated that townships 1 (Figure 11(G)) and 49 (Figure 11(H)) have the same source of imported cases.

**Figure 12:**
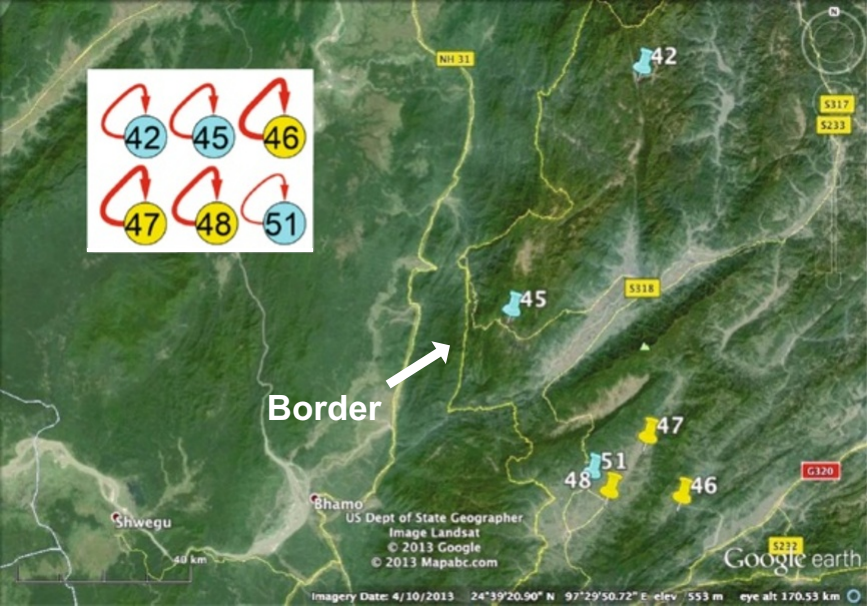
**Townships 42, 45, 46, 47, 48 and 51 are located adjacent to the border between China and Myanmar.** Therefore, their self-connected edges are possibly due to the imported cases.

*Self-Connected Nodes:* As mentioned previously, the external influence component is merged with the internal transmission component. Therefore, these inferred self-connected edges may represent either of these two components, or their combination. Here, we take one group of nodes as an illustrative example. For townships 42, 45, 46, 47, 48, and 51, it is obvious that the endemic disease cases are most likely to be caused by imported cases, because they are located at the border between China and Myanmar (Figure 12). Figure 11(J)-(L) also validate that their reported cases appear consecutively but are not similar with each other.

There are 47 rather than 51 townships in the inferred malaria transmission network. The four missing nodes have neither self-connected edges nor neighborhood-connected edges. The sum of their infection cases is 81, which is a very small proportion of all the infection cases. Therefore, we think their disease transmission dynamics are primarily accidentally imported cases. It seems that although some townships have similar temporal trends, they are not connected, for example, townships 18 (Figure 11(E)) and 50 (Figure 11(L)). The reason could be the choices of both the time window and the time lag. However, because this real-world dataset is very sparse, it is often difficult to choose the right values. In addition, although some townships are located very close to each other, and on the same rivers, they are not connected within the inferred malaria transmission network; for example, townships 34 and 39 in Figure 9 are not connected because their transmission pathways are not significant or their malaria endemics are mainly affected by the imported cases that disguise the impact of the other factors. To interpret them, currently available information about transportation, rivers, and geographical locations may not be adequate, as the transmission pathways are the *comprehensive results of all impact factors*. Moreover, the roads that are locally formed and managed are not displayed in the map, and they may play significant roles in malaria transmission. Missing reports and data sparsity may also affect the results. However, our method can still detect some hidden connections that may draw the attention of policy makers.

## Discussion

There are two key control parameters that play significant roles in the inference results of NetEpi. One is the time window that is used in the partial correlation networks, and the other is the number of observations needed to accurately infer the disease transmission networks. In the following, the influences of those two parameters are discussed individually.

To construct a partial correlation network, it is necessary to select an appropriate time window. Based on a real-world situation, time windows of one day, one week, two weeks, three weeks, one month, five weeks, and one and a half months are selected. In addition, a measurement is defined to evaluate the results:
21

where *s* is the number of edges appearing in both the ground-truth network *G* and the partial correlation network *G*^*p*^, |*E*| is the number of edges in *G*, |*E*^*p*^| is the number of edges contained in *G*^*p*^, and *tw* refers to the selected time window. It is desirable that *G*^*p*^ contains more edges that appear in *G*, and less edges that do not appear in *G*, therefore we use *m*_*tw*_ to measure the trade-off between *s* and |*E*^*p*^|. Based on the experiments and theoretical analysis, the ranges of *s* and |*E*^*p*^| are as follows:
22

Therefore, the value of *m*_*tw*_ increases as *s* increases or |*E*^*p*^| decreases. The ideal case is *s*=|*E*^*p*^|=|*E*|, so that *m*_*tw*_ significantly approximates 1. It should be noted that when *s* approximates or equals |*E*|, *m*_*tw*_ approximates 1 as well, and even |*E*^*p*^| is very large (but still much smaller than |*E*|^2^). Here such cases are not punished, as finding all the ground-truth edges, or the majority of them, is more important than the constructed partial correlation network with a larger size.

**Figure 13:**
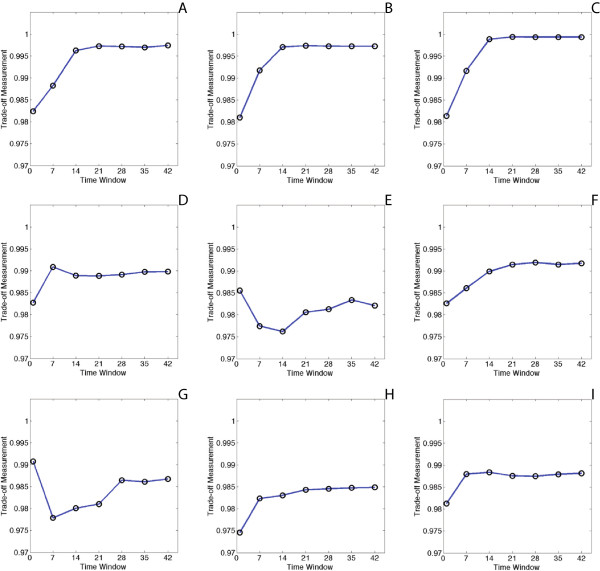
**Sensitivity analysis for the choice of time window on synthetic networks with different sizes and topologies.** The horizontal axis is the selected time window with the unit of day. The vertical axis is the measurement value of *m*_*tw*_ computed from Eq. 21. **(A) - (C)** show core-periphery networks with size of 64 nodes with 100 edges, 128 nodes with 180 edges, and 256 nodes with 350 edges. **(D) - (F)** show hierarchical community networks with size of 64 nodes with 100 edges, 128 nodes with 180 edges, and 256 nodes with 350 edges. **(G) - (I)** show random graphs with size of 64 nodes with 100 edges, 128 nodes with 180 edges, and 256 nodes with 350 edges.

For all the 24 synthetic transmission networks, we take the individual average values of the analyzed results of the 10 independent datasets. Based on the results shown in Figure 13, the relationships between trade-off measurement *m*_*tw*_ and time window *tw* are categorized into four classes.

***“N” Shape:*** Examples of this type of relationship are shown in Figure 13(D). The trade-off measurement value in such case usually achieves the maximum at a time window with less or moderate values, for example, 7 or 14 days. The partial correlation networks also contain fewer edges under such a time window. *m*_*tw*_ decreases at the beginning because the increasing rate of *s* is slow compared to the fast increasing rate of |*E*^*p*^|. *m*_*tw*_ gradually increases later because stronger correlations are identified under the conditions of increasing time window values.

***“S” Shape:*** Examples of this type of relationship are Figure 13(A), (B), (C), (F), (H) and (I). As the length of the time window increases, more edges in the ground-truth networks appear in the partial correlation networks. The correlations of these edges are consolidated as the time-series data are smoothed. At a given point, for example, a time window of 14 days, the majority of the strong correlations have been identified, so that even as the length of time window continues to increase, the number of strongly correlated edges remains stable.

***“V” Shape:*** Examples of this type of relationship are Figure 13(E) and (G). In such cases, the trade-off measurement value reaches the maximum at the very beginning (*tw* = 1), then decreases dramatically to a valley, and increases afterwards. The climax at the start is caused by the low values of both *s* and |*E*^*p*^|. A proportion of ground-truth edges have not been found out when the time window is equal to one day. Moreover, the sizes of the corresponding partial correlation networks are also small. As in the “N” shape, *m*_*tw*_ decreases to a valley because the increasing rate of *s* is slow compared to the fast increasing rate of |*E*^*p*^|. The subsequent increase is the same as in the “N” shape.

Another important control parameter is the number of observations (size of surveillance dataset), which is the parameter *M*. Intuitively, the more data there are, the better the inferred results should be. However, it is usually difficult to obtain complete and sufficient surveillance data because of missing reports, immature surveillance systems, etc. In addition, although a huge amount of data can be collected, big data still poses challenges for both data storage and data analysis. Consequently, experiments testing the influence of the size of the surveillance dataset on the accuracy of NetEpi are conducted.

If the size of the surveillance dataset is much smaller than the length of the time window that NetEpi uses, the construction of the partial correlation networks will fail. Therefore, this research assumes that the size of surveillance dataset should be at least larger than the length of the time window. Specifically, the detailed relationships between the size of surveillance data *M*, length of selected time window *tw*, number of network nodes *N* and the scale parameter *φ* should be as follows:
23

The left-hand side of the above equation is the size of the time-series dataset after smoothing it under time window *tw*. The right-hand side is the size of the available surveillance dataset to be tested. Obviously, this criteria guarantees that no matter how long the selected time window is, given a target scale related to the number of network nodes, it is often possible to find a lower bound for the surveillance data that will ensure that the partial correlation analysis is workable. For example, given a network with 128 nodes (*N* = 128) and a time window of 35 (*tw* = 35), if NetEpi is performed when the surveillance dataset is almost half the size (*φ* = 2) of the number of nodes, then the size of the training surveillance dataset should at least be 98.

**Figure 14:**
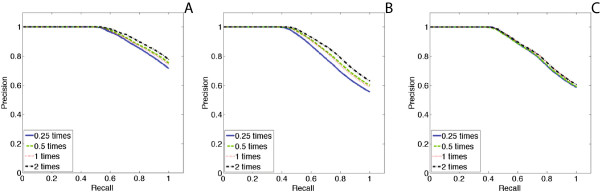
**Sensitivity analysis for the choice of observation or surveillance data with different size.** **(A) - (C)** show the results of networks with different topologies but the same size of 128 nodes and 200 edges. Network in **(A)** is a core-periphery network. Network in **(B)** is a hierarchical community network. Network in **(C)** is a random graph. The curve with the size of a quarter of the number of network nodes is displayed as a blue solid line. The curve with the size of a half the number of network nodes is displayed as a green dashed line. The curve with the size of the same number of network nodes is displayed as a red dotted line. The curve with the size of two times the number of network nodes is displayed as a black dash-dot line.

**Figure 15:**
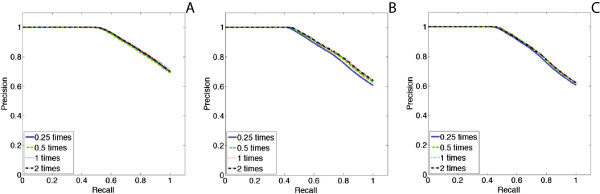
**Sensitivity analysis for the choice of observation or surveillance data with different size.** **(A) - (C)** show the results of networks with different topologies but the same size of 256 nodes and 350 edges. Network in **(A)** is a core-periphery network. Network in **(B)** is a hierarchical community network. Network in **(C)** is a random graph. The curve with the size of a quarter of the number of network nodes is displayed as a blue solid line. The curve with the size of a half the number of network nodes is displayed as a green dashed line. The curve with the size of the same number of network nodes is displayed as a red dotted line. The curve with the size of two times the number of network nodes is displayed as a black dash-dot line.

Figures 14 and 15 show the results of experiments for six networks with different topologies (core-periphery networks, hierarchical community networks, and random graphs) and sizes (128 nodes with 200 edges and 256 nodes with 350 edges). For each network, different sizes of surveillance dataset are tested independently. All of them are tested under the time window of 35. The scale parameter *φ* is set to equal to 4, 2, 1 and 0.5, as shown in the precision-recall curves with the legends 0.25, 0.5, 1 and 2 times, respectively.

In all these experiments, although less surveillance data may bias the accuracy of NetEpi, the bias is not significantly obvious, even in Figure 15(B), as the missing data is not considered during the generation of the synthetic surveillance data. These results confirm that NetEpi can accurately find and estimate those edges that play important roles in disease transmission, even given minimal surveillance data.

## Conclusions

This research bridges the gap between theoretical studies of disease transmission networks and real-world infectious disease transmission, by inversely inferring hidden disease transmission networks using only surveillance data. Specifically, it addresses this problem at a metapopulation level, which is more meaningful and practical for front-line practitioners and policy makers. To achieve this goal, a network inference method called NetEpi is developed. The proposed method and the experimental results provide policy makers with insights into discovering hidden transmission pathways among subpopulations and optimizing limited resources when implementing intervention strategies. In addition, this novel tool can be implemented as a part of surveillance-response system to actively detect and monitor low-transmission patterns [37].

The current version of NetEpi does not consider the detailed impact factors of a specific disease. That is to say, the inferred disease transmission networks are comprehensive and abstract networks that integrate all the impact factors. Taking the inferred malaria transmission network as an example, the inferred edges can be interpreted as geographical locations, convenient traffic routes, suitable habitats for the vector, etc. Therefore, to investigate the transmission pathways in more detail, and to find out the exact interpretations for the inferred edges, it will be necessary to build specific transmission models for different diseases. Moreover, various impact factors should be carefully integrated.

Another direction for future work is to infer dynamic disease transmission networks. Currently, the assumption is that the hidden disease transmission networks do not change within a prefixed time period. However, in reality, the network may change as impact factors change over time. Therefore, inferring dynamic disease transmission networks is useful over a long-time scale, which is also more helpful for policy makers to design long-term intervention strategies.

Finally, the current back-tracking technique rolls back the optimization procedure roughly rather than smoothly, and converges to either the local optimum or the global optimum. Therefore, future work should modify this technique to improve precision.

## References

[1] Eames KTD, Keeling MJ (2003). **Contact tracing and disease control**. Proc R Soc Lond B Biol Sci.

[2] Newman ME (2002). **Spread of epidemic disease on networks**. Phys Rev E.

[3] Riley S (2007). **Large-scale spatial-transmission models of infectious disease**. Science.

[4] Eubank S, Guclu H, Anil Kumar VS, Marathe MV, Srinivasan A, Toroczkai Z, Wang N (2004). **Modelling disease outbreaks in realistic urban social networks**. Nature.

[5] Pastor-Satorras R, Vespignani A (2001). **Epidemic dynamics and endemic states in complex networks**. Phys Rev E.

[6] Keeling JM, Eames TDK (2005). **Networks and epidemic models**. J R Soc Interface.

[7] Salathé M, Jones JH (2010). **Dynamics and control of diseases in networks with community structure**. PLoS Comput Biol.

[8] Hollingsworth TD, Ferguson NM, Anderson RM (2006). **Will travel restrictions control the international spread of pandemic influenza?**. Nat Med.

[9] Sebastian F, Marcel S, Vincent JAA (2010). **Modelling the influence of human behaviour on the spread of infectious diseases: A review**. J R Soc Interface.

[10] Bajardi P, Poletto C, Ramasco JJ, Tizzoni M, Colizza V, Vespignani A (2011). **Human mobility networks, travel restrictions, and the global spread of 2009 h1n1 pandemic**. PLoS ONE.

[11] Hufnagel L, Brockmann D, Geisel T (2004). **Forecast and control of epidemics in a globalized world**. Proc Natl Acad Sci U S A.

[12] Liu J, Yang B, Cheung W, Yang G (2012). **Malaria transmission modelling: a network perspective**. Infectious Diseases Poverty.

[13] Leventhal GE, Kouyos R, Stadler T, von Wyl V, Yerly S, Böni J, Cellerai C, Klimkait T, Günthard HF, Bonhoeffer S (2012). **Inferring epidemic contact structure from phylogenetic trees**. PLoS Comput Biol.

[14] Gomez-Rodriguez M, Leskovec J, Krause A (2010). **Inferring networks of diffusion and influence**. Proceedings of the 16th ACM SIGKDD International Conference on Knowledge Discovery and Data Mining. KDD ’10.

[15] Kempe D, Kleinberg J, Tardos E (2003). **Maximizing the spread of influence through a social network**. Proceedings of the 9th ACM SIGKDD International Conference on Knowledge Discovery and Data Mining. KDD ’03.

[16] Myers S, Leskovec J, Lafferty J, Williams CKI, Shawe-Taylor J, Zemel RS, Culotta A (2010). **On the convexity of latent social network inference**. Advances in Neural Information Processing Systems 23.

[17] Myers SA, Zhu C, Leskovec J (2012). **Information diffusion and external influence in networks**. Proceedings of the 18th ACM SIGKDD International Conference on Knowledge Discovery and Data Mining. KDD’12.

[18] Teunis P, Heijne JCM, Sukhrie F, van Eijkeren J, Koopmans M, Kretzschmar M (2013). **Infectious disease transmission as a forensic problem: Who infected whom?**. J R Soc Interface.

[19] Arino J, Ma Z, Zhou Y, Wu J (2009). **Diseases in metapopulations**. Modeling and Dynamics of Infectious Diseases. Series in Contemporary Applied Mathematics, Volume 11.

[20] Colizza V, Vespignani A (2008). **Epidemic modeling in metapopulation systems with heterogeneous coupling pattern: Theory and simulations**. J Theor Biol.

[21] Ajelli M, Goncalves B, Balcan D, Colizza V, Hu H, Ramasco J, Merler S, Vespignani A (2010). **Comparing large-scale computational approaches to epidemic modeling: agent-based versus structured metapopulation models**. BMC Infect Dis.

[22] Lentz HHK, Selhorst T, Sokolov IM (2012). **Spread of infectious diseases in directed and modular metapopulation networks**. Phys Rev E.

[23] Ndeffo Mbah ML, Gilligan CA (2011). **Resource allocation for epidemic control in metapopulations**. PLoS ONE.

[24] Yang X, Liu J, Cheung WKW, Zhou X-N (2014). **Inferring metapopulation based disease transmission networks**. Advances in Knowledge Discovery and Data Mining. Lecture Notes in Computer Science, Volume 8444.

[25] Yang X (2014). Inferring disease transmission networks.

[26] Shang C-S, Fang C-T, Liu C-M, Wen T-H, Tsai K-H, King C-C (2010). **The role of imported cases and favorable meteorological conditions in the onset of dengue epidemics**. PLoS Negl Trop Dis.

[27] Dénes A, Kevei P, Nishiura H, Röst G (2013). **Risk of infectious disease outbreaks by imported cases with application to the european football championship 2012**. Int J Stochastic Anal.

[28] Yuan Y, Li C-T, Windram O (2011). **Directed partial correlation: Inferring large-scale gene regulatory network through induced topology disruptions**. PLoS ONE.

[29] Lasserre J, Chung H-R, Vingron M (2013). **Finding associations among histone modifications using sparse partial correlation networks**. PLoS Comput Biol.

[30] Wipf DP, Rao BD (2004). **Sparse bayesian learning for basis selection**. IEEE Trans Signal Process.

[31] Tipping ME (2001). **Sparse bayesian learning and the relevance vector machine**. J Mach Learn Res.

[32] Tzikas DG, Likas CL, Galatsanos NP (2009). **Sparse bayesian modeling with adaptive kernel learning**. IEEE Trans Neural Netw.

[33] Leskovec J, Faloutsos C (2007). **Scalable modeling of real graphs using kronecker multiplication**. Proceedings of the 24th International Conference on Machine Learning, ICML ’07.

[34] WHO (2012). World Malaria Report 2012.

[35] Brasil P, de Pina Costa A, Pedro R, da Silveira Bressan C, da Silva S, Tauil P, Daniel-Ribeiro C (2011). **Unexpectedly long incubation period of plasmodium vivax malaria, in the absence of chemoprophylaxis, in patients diagnosed outside the transmission area in brazil**. Malaria J.

[36] Hulden L, Hulden L, Heliovaara K (2008). **Natural relapses in vivax malaria induced by anopheles mosquitoes**. Malaria J.

[37] Zhou X-N, Bergquist R, Tanner M (2013). **Elimination of tropical disease through surveillance and response**. Infectious Diseases Poverty.

